# Psychology of Aging Podcast: Public Education Tool for Sharing Evidence-Based Mental Health and Aging Information

**DOI:** 10.1093/geroni/igab046.1943

**Published:** 2021-12-17

**Authors:** Regina Koepp

**Affiliations:** Center for Mental Health & Aging, Brookhaven, Georgia, United States

## Abstract

Psychology of Aging Podcast, created and hosted by Dr. Regina Koepp, Clinical Geropsychologist, is the first podcast of its kind devoted solely to mental health and aging. The goal of the Psychology of Aging podcast is to facilitate access to information and education about mental health and brain health among older adults with the hope of de-stigmatizing mental health care for older adults, reducing ageism, highlighting diversity, and promoting access to mental health and dementia care. The format includes a combination of expert interviews and “solo-casts”. Topics range from depression and suicide prevention to Alzheimer’s Disease and related dementias to the unique needs of LGTBQ older adults and caregivers to health disparities experienced by African American and Latin-X communities related to dementia and the COVID-19 pandemic. During this session, Dr. Koepp will discuss the role podcasts play in public education and share tips for starting an evidence-based podcast.

